# Study of Low-Velocity Impact Behavior of Hybrid Fiber-Reinforced Metal Laminates

**DOI:** 10.3390/polym16020173

**Published:** 2024-01-06

**Authors:** Yuting Fang, Dongfa Sheng, Zhongzhao Lin, Peng Fei

**Affiliations:** 1 School of Civil Engineering, Southwest Forestry University, Kunming 650224, China; fangyuting@swfu.edu.cn (Y.F.); linzhongzhao@swfu.edu.cn (Z.L.); 2 Key Laboratory of Forest Disaster Warning and Control of Yunnan Province, Southwest Forestry University, Kunming 650224, China; 3 School of Resources, Environment and Safety Engineering, University of South China, Hengyang 421001, China; fp1798547531@126.com

**Keywords:** fiber metal laminates, hybrid fiber, low-velocity impact, numerical simulation, low-velocity impact behavior, damage mode

## Abstract

In this paper, the low-velocity impact behavior and damage modes of carbon/glass-hybrid fiber-reinforced magnesium alloy laminates (FMLs-H) and pure carbon-fiber-reinforced magnesium alloy laminates (FMLs-C) are investigated using experimental, theoretical modeling, and numerical simulation methods. Low-velocity impact tests were conducted at incident energies of 20 J, 40 J, and 60 J using a drop-weight impact tester, and the load–displacement curves and energy–time curves of the FMLs were recorded and plotted. The results showed that compared with FMLs-C, the stiffness of FMLs-H was slightly reduced, but the peak load and energy absorption were both greatly improved. Finally, a finite element model based on the Abaqus-VUMAT subroutine was developed to simulate the experimental results, and the damage modes of the metal layer, fiber layer, and interlayer were observed and analyzed. The experimental results are in good agreement with the finite element analysis results. The damage mechanisms of two kinds of FMLs under low-velocity impacts are discussed, providing a reference for the design and application of laminates.

## 1. Introduction

Fiber metal laminates (FMLs) are composite materials made by laying up reinforcing fibers and metal sheets alternately and preparing them under certain temperature and pressure. As it combines the excellent mechanical properties of fiber and metal materials, it has the advantages of high specific strength, high specific stiffness, resistance to impact damage, high temperature and corrosion resistance, etc., and is widely used in aerospace, electronic information, and the military industry, and other fields [1,2,3,4,5]. FMLs are not susceptible to chemical corrosion, and their mechanical properties do not change under a certain pressure. The mechanical properties of FMLs at specific temperature are related to the metal used, and degradation of the mechanical properties of FMLs occurs when the temperature exceeds the threshold value that the metal can withstand.

It is often unavoidable for composite components to be damaged by low-velocity impacts in the process of its use, for example, when a car is hit by another vehicle while driving, when an airplane is hit by a flock of birds during flight, or when a ship is hit by a floating object while sailing, and so on [6,7,8,9,10,11]. The occurrence of such events will cause structural damage to FMLs, thereby reducing their service life and greatly weakening their load-carrying capacity. Therefore, it is particularly important to investigate the mechanical behavior of FMLs under low-velocity impact.

In recent years, many researchers have investigated the mechanical behavior of FMLs under impact loading by means of experiments and finite element simulations. Jaroslaw et al. [12] compared the mechanical behavior of aluminum-based glass fiber laminates and aluminum-based carbon fiber laminates under low-velocity impacts in terms of damage area and damage depth. Research showed that the important damage mode of FMLs is interfacial debonding. Aluminum-based carbon fiber plywood has a higher tendency to perforate, and the two have completely different energy absorption modes. Yao et al. [13] investigated the mechanical behavior and damage mechanism of aluminum-based composites under low-velocity impact using different shapes of punches, and the results showed that the damage mode of FMLs under the impact of conical punches was mainly petaling crack, and the damage mode under the impact of flat-shaped punches was mainly smooth perforation. The experimental results were verified by establishing a finite element model, and it was found that the impact load–time curves and damage morphology of the two could be better coincided. Lee et al. [14] found that FMLs fabricated using autoclave molding have higher strength and stiffness, better impact resistance, and lighter mass compared to conventional FMLs through drop-weight impact tests. They also investigated the crack extension and damage modes of FMLs with different ply sequences, and the results showed that the direction of crack extension was affected by fiber orientation. Some scholars have investigated the mechanical behavior of FMLs under multiple impacts. Pai et al. [15] incorporated aramid—epoxy, ultra-high molecular weight polyethylene—epoxy, and paperboard—epoxy as interlayers, and observed and analyzed the deformation distributions and damage modes of FMLs under primary and secondary impacts. It was concluded that the FMLs incorporating the paperboard layer had smaller deformations compared to those without the paperboard layer. Moreover, it was found that the addition of high acoustic impedance metal panels could enhance the impact resistance of FMLs to a certain extent. The established numerical model can predict the deformation profile of the backplate of FMLs more accurately.

Hybrid composites are composite materials made by combining two or more fibers in the same matrix, in which the fibers can be selected from natural fibers, artificial fibers, or synthetic fibers, and various fibers can be arranged in different layup sequences to optimize the performance of the composites. Compared with single-fiber-reinforced composites, hybrid composites have the advantages of reducing weight, lowering production costs, improving strength and stiffness, and improving impact resistance and fatigue resistance, which are widely used in aerospace, construction industry, health care, and other fields [16,17,18,19,20].

Several scholars have successfully used hybridization to enhance the impact resistance of homogeneous laminates. For research on hybrid composites, at the beginning, most of the literature mentioned the combination of carbon/aramid and carbon/polyethylene to enhance the impact resistance of the laminate. As the environmental requirements became more stringent, the research on basalt fibers gradually increased. Sarasini et al. [21,22] investigated the effect of basalt hybridization on the impact properties of aramid fiber laminates and carbon fiber/epoxy laminates and performed four-point bending tests after impact. The results showed that the hybrid laminates have superior energy absorption and flexural behavior and better damage tolerance than the homogeneous laminates. With the development of hybrid composites, some scholars began to combine hybrid fibers with FMLs. Megeri et al. [23] investigated the mechanical properties of glass/carbon fiber-reinforced aluminum alloy laminates under low-velocity impacts and found that hybrid fiber-reinforced aluminum alloy laminates have better impact resistance and smaller central deflection compared to pure glass-fiber-reinforced aluminum alloy laminates. Kazemi et al. [24] hybridized carbon and UHMWPE fibers and investigated the mechanical properties of fiber-reinforced titanium alloy laminates under low-velocity impact. It was found that FMLs with added UHMWPE fibers had better toughness and ductility and performed better at LVI compared to pure carbon fiber reinforced titanium laminates. Hussain et al. [25] used the matrix and hybrid fibers as variables to investigate the low-velocity impact behavior of hybrid fiber-reinforced aluminum alloy laminates. They found that FMLs have better toughness and higher impact resistance when using Polyvinyl Butyral as the matrix, while epoxy-based FMLs have poorer impact resistance. A comparison of different types of hybrid reinforcements reveals that aramid/jute hybrid fibers have higher deformation resistance and show better overall LVI performance compared to carbon/jute hybrid fibers.

At present, many scholars usually choose an aluminum alloy as the metal layer when studying the mechanical properties of FMLs, while research on magnesium alloy, especially on hybrid fiber-reinforced magnesium alloy laminates is less common. In this paper, the mechanical properties of pure carbon-fiber-reinforced magnesium alloy laminates and carbon and glass hybrid fiber-reinforced magnesium alloy laminates under low-velocity impact are investigated through experiments, theoretical models, and numerical simulations. Firstly, the displacement–load curves and energy–time curves of FMLs were obtained through experiments, and then a finite element model is established based on Abaqus-VUMAT subroutine to observe and analyze the damage modes and damage morphology of the metal layer, the fiber layer, and the interlayer, and the experimental results were compared with those of the finite element analysis. The above study applied hybrid fibers to FMLs and investigated their impact properties and damage mechanisms, filling a gap in the field. The established finite element model can better predict the mechanical properties and damage patterns of FMLs, which contributes to the application and development of FMLs in practical engineering.

## 2. Specimen Preparation and Experimental Design

### 2.1. FMLs Specimen Preparation

**Figure 1:**
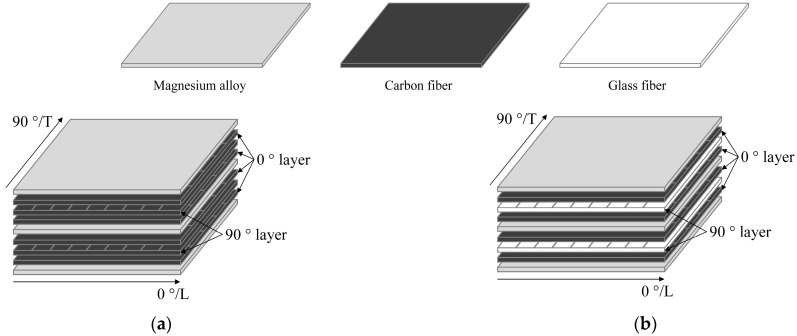
Ply design of FMLs: (**a**) FMLs-C; (**b**) FMLs-H.

The specimens are made of an AZ31B magnesium alloy sheet (Dongguan Lianwei Magnesium Alloy Material Ltd., Dongguan, China), carbon fiber prepregs (T700-12K, Jiangyin Tiangui New Material Technology Ltd., Wuxi, China), and glass fiber prepregs (Nanjing Xinhe Composites Co., Nanjing, China) laid alternately. The type of epoxy resin and curing agent is BE188EL/AM838, and the ply design is shown in Figure 1. The codes of each FML specimen and the related information are presented in Table 1. The thickness of the magnesium alloy sheets is 0.5 mm, the thickness of the fiber prepregs is 0.15 mm, and the average thickness of the specimens is 2.4 mm. The layup sequence of the FMLs is [Mg/0°/90°/0°/Mg/0°/90°/0°/Mg°].

To facilitate a better bonding between the magnesium alloy layer and the fiber prepreg, the magnesium alloy sheet was subjected to surface modifications. First, silicon carbide abrasive paper of 120# grit was used to sand the sheet to roughen the surface. After degreasing with acetone, the sheet was then treated with acid washing, alkaline washing, and water washing to remove surface oxides and impurities [26]. Finally, the sheet was modified with potassium permanganate solution. After the treatment was completed, the surface of the specimen was cleaned and dried.

**Figure 2:**
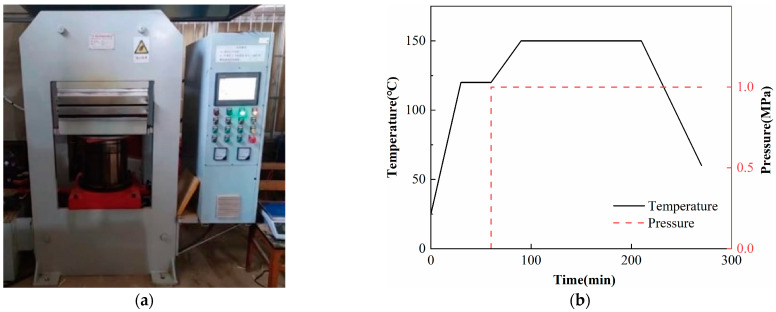
Hot-pressing process of FMLs: (**a**) Hot press machine; (**b**) curing process.

The surface-pretreated metal plate and fiber prepreg were neatly placed into the mold, and the specimens were hot-pressed into shape using a Qingdao Huabo hot press machine, as shown in Figure 2a. The hot press machine was first warmed up from room temperature to 120 °C for half an hour, and then warmed up to 150 °C. Then, 1 MPa of pressure was applied to cure the specimens at 150 °C for 2 h. The curing process is shown in Figure 2b. After the specimens were cooled to room temperature naturally, they were cut into rectangular shapes of 150 mm × 100 mm.

### 2.2. Low-Velocity Impact and Damage Assessment

**Figure 3:**
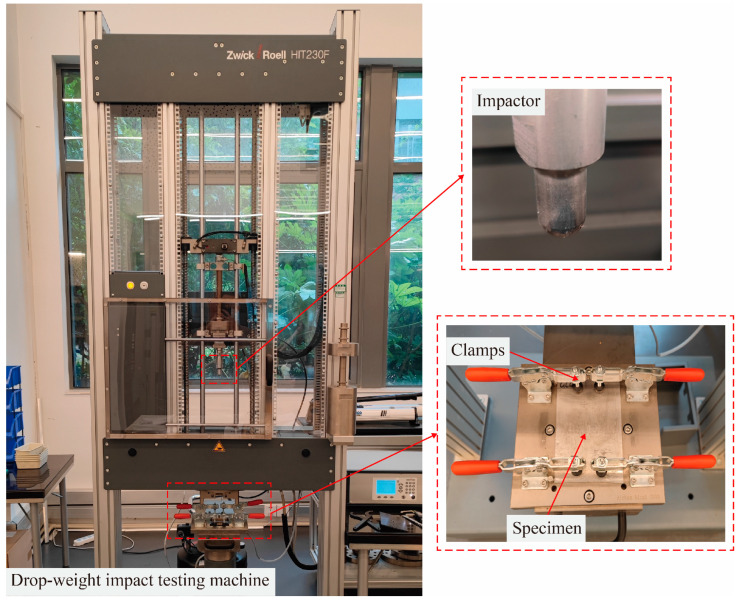
Low-velocity impact test at different energies.

The drop-weight impact testing machine HIT 230F (Zwick Roell Ltd., Ulm, Germany), which conforms to ASTM D7136 [27], was employed to perform a low-speed impact on the specimens at an incident energy of 40 J, 60 J, and 80 J, respectively, as shown in Figure 3. To ensure the specimens are centered, mechanical clamps were used to fix the four sides of the specimens. The punch has a hemispherical shape, a diameter of 16 mm, and a weight of 200 g. To ensure the accuracy of the test, five specimens of each type were tested at each incident energy.

## 3. Finite Element Modeling

Using the finite element software Abaqus/Explicit 2021 (version number: 6.423.0.0), the damage modes and failure behavior of FMLs under impact loading are investigated. The FMLs consisted of the metal layer, the fiber/epoxy layer, and the interlayer. Damage models are built for each layer, and failure modes for each layer are defined by either the software’s own procedures or the Abaqus-VUMAT subroutine.

### 3.1. Metal Layer Damage Model

The Johnson–Cook (J-C) constitutive model has a simple form and is easy for engineering applications, in addition to its ability to consider the relationship between stress and strain, strain rate, and temperature, is widely used in impact dynamics. The general expression of the J-C constitutive model [28] is

(1) $$\sigma =\left({A+B\varepsilon }^{n}\right)\left(1+C\ln{\dot{\varepsilon }}^{*}\right)\left(1-T^{*m}\right)$$

where ε means the equivalent plastic strain, and ${\dot{\varepsilon }}^{*}=\dot{\varepsilon }/\varepsilon_{0}$ means the strain rate. $T^{*}$ means the homologous temperature. *A*, *B*, *C*, n, and m are the constitutive model parameters, which are derived by quasi-static tensile tests. The values of the above parameters can be found in Table 2.

To further investigate the damage process of the metal layer, the J-C constitutive model is extended to include a fracture model based on cumulative damage, with the following expression:

(2) $$D=\Sigma \frac{\Delta \varepsilon }{\varepsilon_{f}}$$

where D means the damage factor of the material. When D=1, the material is completely destroyed. Δε is the equivalent plastic strain increment. $\varepsilon_{f}$ is the equivalent plastic fracture strain at current time step, and the general expression of $\varepsilon_{f}$ is

(3) $$\varepsilon_{f}=\left[D_{1}+D_{2}\exp D_{3}\sigma^{*}\right]\left[1+D_{4}\ln{\dot{\varepsilon }}^{*}\right]\left[1+D_{5}T^{*}\right]$$

(4) $$\sigma^{*}=\frac{\sigma_{m}}{\bar{\sigma }}$$

where $D_{1}$~$D_{5}$ are the failure model parameters. $\sigma^{*}$ is the stress triaxiality, $\bar{\sigma }$ represents the Von Mises equivalent stress, and $\sigma_{m}$ means the average of the three normal stresses. The failure model parameters of the AZ31B magnesium alloy can be found in Table 3 [29].

### 3.2. Fiber/Epoxy Layer Damage Model

The main failure modes of the fiber/epoxy layer include fiber failure, matrix failure, and laminate delamination failure. The Hashin failure criterion [30,31] and Yeh delamination failure criterion [32] are chosen for modeling in this paper due to their simple form. The five failure modes considered in the failure criterion are shown below.

Fiber tensile failure ($\sigma_{11}\;\geq \;0$)

(5) $${\left(\frac{\sigma_{11}}{X_{T}}\right)}^{2}+{\left(\frac{\tau_{12}}{S_{12}}\right)}^{2}+{\left(\frac{\tau_{13}}{S_{13}}\right)}^{2}\;\geq \;1$$

Fiber compressive failure ($\sigma_{11}\;<\;0$)

(6) $${\left(\frac{\sigma_{11}}{X_{C}}\right)}^{2}\;\geq \;1$$

Matrix tensile failure ($\sigma_{22}+\sigma_{33}\;\geq \;0$)

(7) $${\left(\frac{\sigma_{22}+\sigma_{33}}{Y_{T}}\right)}^{2}+\frac{1}{S_{23}^{2}}\left(\tau_{23}^{2}-\sigma_{22}\sigma_{33}\right)+{\left(\frac{\tau_{12}}{S_{12}}\right)}^{2}+{\left(\frac{\tau_{13}}{S_{13}}\right)}^{2}\;\geq \;1$$

Matrix compressive failure ($\sigma_{22}+\sigma_{33}\;<\;0$)

(8) $$\left(\frac{\sigma_{22}+\sigma_{33}}{Y_{T}}\right)\left[{\left(\frac{Y_{C}}{2S_{23}}\right)}^{2}-1\right]+\frac{{\left(\sigma_{22}+\sigma_{33}\right)}^{2}}{4S_{23}}+\frac{1}{S_{23}^{2}}\left(\tau_{23}^{2}-\sigma_{22}\sigma_{33}\right)+{\left(\frac{\tau_{12}}{S_{12}}\right)}^{2}+{\left(\frac{\tau_{13}}{S_{13}}\right)}^{2}\;\geq \;1$$

Laminate delamination failure ($\sigma_{33}\;\geq \;0$)

(9) $${\left(\frac{\sigma_{33}}{Z_{T}}\right)}^{2}+{\left(\frac{\tau_{13}}{S_{13}}\right)}^{2}+{\left(\frac{\tau_{23}}{S_{23}}\right)}^{2}\geq 1$$

In Equations (6)–(9), $X_{C}$, $X_{T}$, $Y_{C}$, $Y_{T}$, $Z_{C}$, and $Z_{T}$ represents the tensile and compressive strength in each direction of the plywood cover, respectively. $\sigma_{\mathrm{ij}}$ and $\tau_{\mathrm{ij}}$ represents the positive and shear stresses in each direction of the plywood, respectively. $S_{\mathrm{ij}}$ is the shear strength in the corresponding plane. In this paper, T700 carbon fiber and S-glass fiber are selected, and the mechanical properties of the two fibers taken from other references as well as suppliers, as shown in Table 4.

When the material satisfies any of these failure modes, experiments have shown that the material does not fail completely; it still has the carrying capacity. In this paper, the stiffness reduction is performed according to the gradual degradation model proposed by Tserpes [33], and the model is shown in Table 5. Based on the above-mentioned failure modes, the Abaqus-VUMAT subroutine is written to simulate the damage of the fiber/resin layer.

### 3.3. Interlayer Damage Model

Using the cohesion zone model (CZM) in Abaqus, the interfacial damage and interlayer damage of the laminate are simulated. This paper uses the Damage for Traction Separation Laws module in Abaqus. The QUADS criterion [34] is chosen to describe the damage initiation process, while the B-K equivalent force fracture criterion [35] is used to describe the damage evolution process. To define the linear-elastic behavior of the element, the traction-separation law built into the software was used. The constitutive model is

(10) $$\sigma =\left[\begin{array}{c} \sigma_{n}\\ \sigma_{s}\\ \sigma_{t} \end{array}\right]=E\varepsilon =\left[\begin{array}{ccc} E_{\mathrm{nn}} & & \\ & E_{\mathrm{ss}} & \\ & & E_{\mathrm{tt}} \end{array}\right]\left[\begin{array}{c} \varepsilon_{n}\\ \varepsilon_{s}\\ \varepsilon_{t} \end{array}\right]$$

where σ and ε are the stress vector and strain vector, respectively. E is the elastic stiffness matrix. The QUADS criterion was used after damage onset, and its general expression is

(11) $${\left\{\frac{\sigma_{n}}{\sigma_{n}^{m}}\right\}}^{2}+{\left\{\frac{\sigma_{s}}{\sigma_{s}^{m}}\right\}}^{2}+{\left\{\frac{\sigma_{t}}{\sigma_{t}^{m}}\right\}}^{2}=1$$

where $\sigma_{n}^{m}$, $\sigma_{s}^{m}$, and $\sigma_{t}^{m}$ represent three modes of fracture strength, respectively. After the laminate begins to delaminate, the B-K (Benzeggagh–Kenane) fracture criterion is introduced to determine the fracture energy release rate $G^{c}$ at the interface of mixed delamination evolution, with a general expression of

(12) $$G^{c}=G_{n}^{c}+\left(G_{s}^{c}-G_{n}^{c}\right){\left(\frac{G_{s}+G_{t}}{G_{n}+G_{s}+G_{t}}\right)}^{\eta }$$

where $G_{n}$, $G_{s}$, and $G_{t}$ represent three modes of strain energy release rates, respectively. $G_{n}^{c}$ and $G_{s}^{c}$ are the corresponding critical strain energy release rates, respectively. η is the correction factor, which is generally taken as 1–2. The values of each factor can be found in Table 6 [36].

### 3.4. Finite Element Model

**Figure 4:**
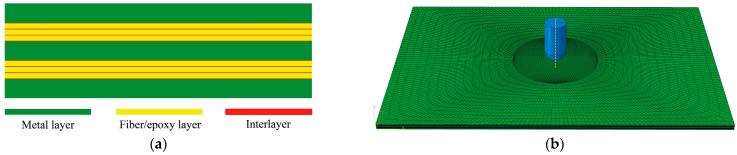
Finite element model of FMLs: (**a**) cross-section of FMLs; (**b**) finite element impact model.

The FMLs finite element model is shown in Figure 4. Both metal and fiber layers are meshed with C3D8R, and interlayer damage is simulated using COH3D8. The contact between the punch and the laminate is set as general contact, the contact property in the normal direction is set to the penalty function contact, the contact property in the tangential direction is set to hard contact, and the coefficient of friction is set as 0.3. In order to ensure the accuracy of the calculation and to make the calculation process more concise, the mesh of the contact area is refined, and the minimum mesh size is 0.5 mm × 0.5 mm. The FML finite element model contains three metal layers, six unidirectionally laid fiber layers, and eight interlayers, with a consistent grid distribution for each individual layer in the laminate.

## 4. Results and Discussion

### 4.1. Low-Velocity Impact Behavior of FMLs

**Figure 5:**
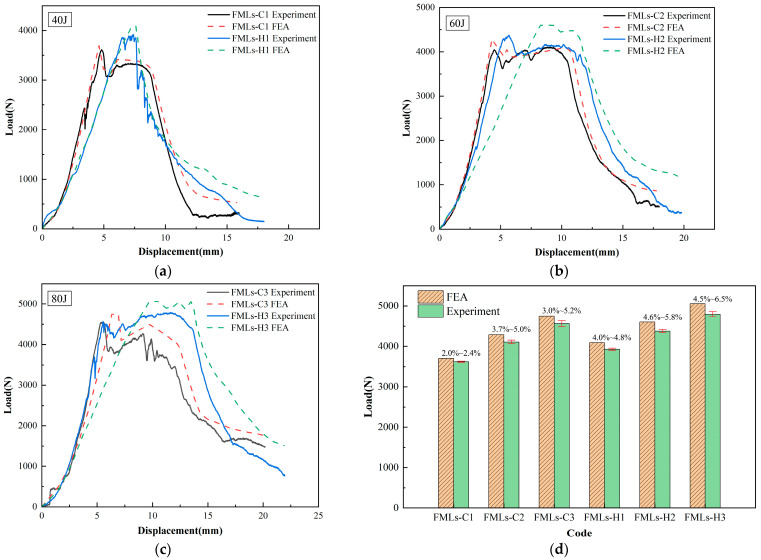
Load–displacement curves of FMLs: (**a**) 40 J; (**b**) 60 J; (**c**) 80 J; (**d**) difference analysis.

Figure 5 shows the experimental results and finite element analysis (FEA) results of the load–displacement curves. From the figure, it can be seen that the curves oscillate significantly near the peak load, which may be due to the impending breakage of the fibers inside the laminates. In comparison with FMLs-H, FMLs-C has a lower peak load (the difference between the two is about 5.1–8.5%), but its displacement is smaller and has better stiffness. As the incident energy increases, the displacement of FMLs is greater, and the damage is more severe. It can be observed that under the effect of a low-velocity impact, the second peak of FMLs-H occurs, while FMLs-C keeps degrading, which may be due to the fact that the hybrid fiber laminates have better mechanical properties and can delay the rate of damage generation. After comparing the experimental results with the FEA results, it is found that the slopes of both are in close agreement at the initial stage. The experimental value of the peak load is slightly lower than the simulated value, which may be due to the unavoidable mechanical damage in the preparation and processing stages of the specimen. At incident energies of 40 J, 60 J, and 80 J, the differences between the experimental and simulated values are 2.0–4.8%, 3.7–5.8%, and 3.0–6.5%, respectively, which indicated that the established finite element model is able to accurately describe the damage behavior of FMLs.

**Figure 6:**
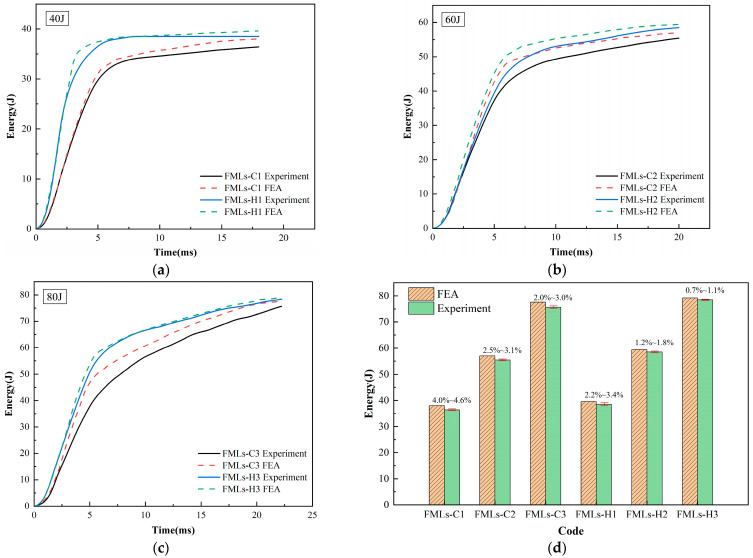
Energy–time curves for FMLs: (**a**) 40 J; (**b**) 60 J; (**c**) 80 J; (**d**) difference analysis.

Figure 6 shows the experimental results and FEA results of the energy–time curves of FMLs. Under a low-velocity impact, the ratio of energy absorption in FMLs increases with increasing energy. The energy absorption capacity of FMLs-H is significantly higher than that of FMLs-C, with a difference of about 3.7–5.7%. By analyzing the experimental results with the FEA results, it can be found that the FML experimental results are slightly lower than the FEA results at different incident energies. At incident energies of 40 J, 60 J, and 80 J, the experimental and simulated values of the absorbed energy of FMLs-C and FMLs-H are in good agreement with each other, with differences of 2.2–4.6%, 1.2–3.1%, and 0.7–3.0%, respectively, which suggests that the established finite element models can accurately predict the experimental results.

### 4.2. Comparison of Finite Element Analysis with Experiment

**Figure 7:**
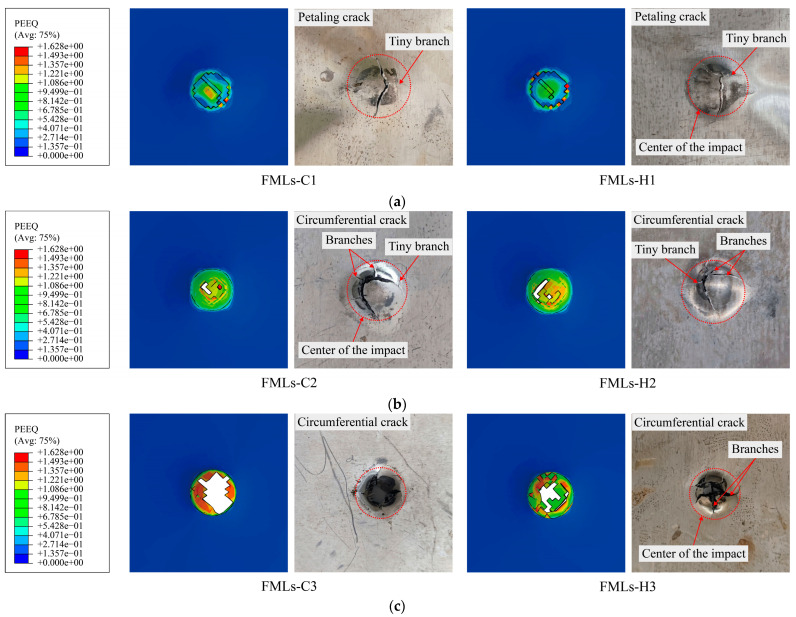
Comparison of damage morphology and experimental of magnesium alloy layers at different incident energies: (**a**) 40 J; (**b**) 60 J; (**c**) 80 J.

Figure 7 shows the equivalent plastic cloud diagrams of magnesium alloy layers at different incident energies and compares them with the experimental results. From the figure, it can be concluded that when the incident energy is 40 J, the damage mode of FMLs is mainly accompanied by petaling cracks with tiny branches. At this time, the cracks on the surface of FMLs are shallow, and no actual notches are observed. With the gradual increase in incident energy, the damage mode of the FMLs changes from dent and shallow crack to penetration mode. When the incident energy is low, due to the greater surface energy of carbon and glass fibers, the interaction force between the two is stronger during the infiltration of the resin and fibers, and adhesion and infiltration can occur spontaneously, which slows down the erosion process of the material to a certain extent. When the incident energy is 60 J, the damage mode of FMLs changes to circumferential crack, and the crack branches near the center of incident begin to increase. When the incident energy is 80 J, the circumferential crack and its branches in FMLs-H3 are seriously extended, and a large notch appears in the impact center, while FMLs-C3 is completely penetrated by the punch and loses its load-bearing capacity. Observing the finite element cloud diagrams and experimental results, it can be found that at the same incident energy, the damage area of FMLs-C is larger than that of FMLs-H, and it is easier to be penetrated.

Since only the damage morphology of the outermost layer could be observed in the experiments, in order to further analyze the damage modes of FMLs, the fiber layers and interlayers of FMLs were simulated using finite element software.

SDVs (Solution-Dependent State Variables) denote the state variable defined in the Abaqus subroutine, where SDV2 refers to fiber damage. Whether the model is damaged or not is controlled by unit deletion; when the damage status reaches 1, unit deletion is performed. After deletion, this unit no longer carries and transmits loads.

**Figure 8:**
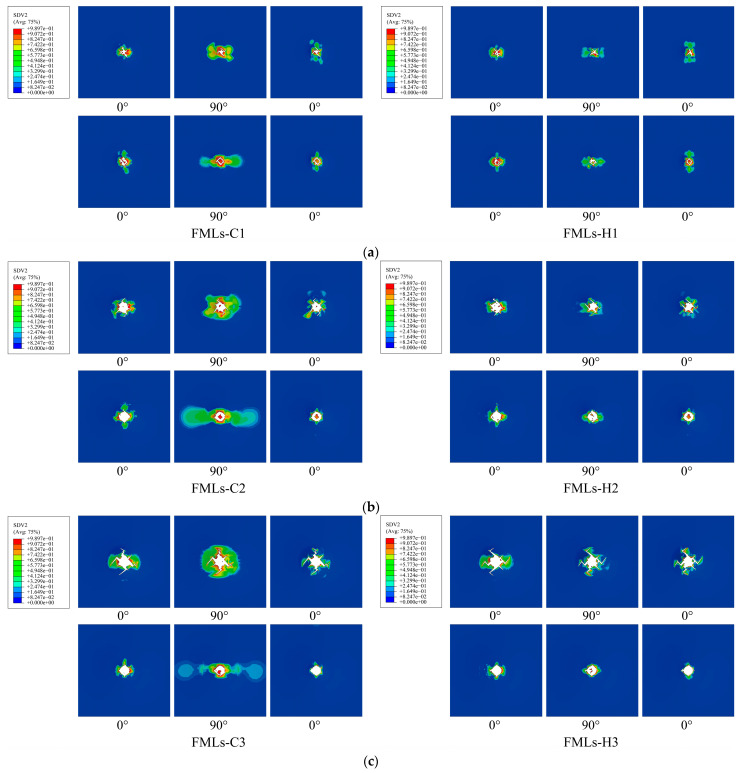
Damage morphology of fiber layers at different incident energies: (**a**) 40 J; (**b**) 60 J; (**c**) 80 J.

The damage morphologies of fiber layers at different incident energies are shown in Figure 8. The observation of the cloud diagram reveals that after impact, the upper fibers are more severely damaged, with more cracks extending from the center of the impact and more units being deleted. In contrast, the lower fibers are less damaged, with unit deletion occurring only in the center, thus acting as a limitation for the punch to fully penetrate the laminate. Comparing FMLs-C and FMLs-H, it can be seen that at the same incident energy, the damage area of FMLs-H is smaller than that of FMLs-C and has better integrity. In comparison with FMLs-C, FMLs-H also suffers from fiber fracture, but the fiber elongation at fracture is smaller, with fewer crack branches, and provides better impact resistance, which may be due to the hybridization of glass fibers in FMLs-H and interaction with carbon fibers. After comparing the damage morphology of FMLs at different incident energies, it can be found that after being subjected to a low-velocity impact, the damage firstly occurs in the center of the fiber layer and gradually extends to the surroundings. More crack branches appeared in the upper fiber layer. With the gradual increase in incident energy, the damage area also gradually expands. When the incident energy reaches 80 J, the FMLs show a penetration phenomenon and basically lose their load-bearing capacity, which also coincides with the phenomenon shown in Figure 7c.

**Figure 9:**
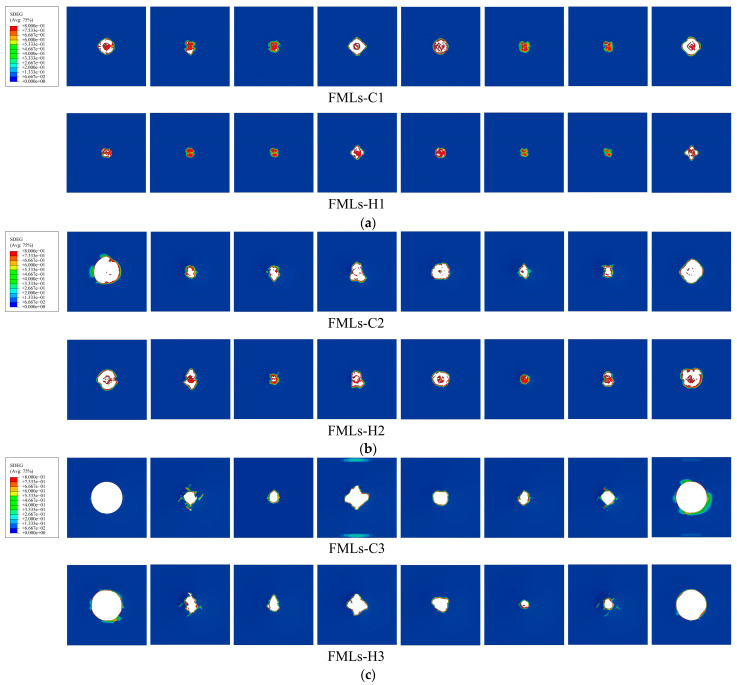
Damage morphology of interlayers under different incident energies: (**a**) 40 J; (**b**) 60 J; (**c**) 80 J.

Figure 9 shows the debonding and damage morphology of the interlayers of FMLs at different incident energies. Observing the debonding of each layer, it can be seen that the debonding between the fiber layers and the metal layers (layers 1, 4, 5, and 8) is more serious compared to the interlayers between the fiber layers (layers 2, 3, 6, and 7). Comparing FMLs-C and FMLs-H, it can be seen that at an incident energy of 40 J, the damage to the interlayers is not serious, and that the interlayers between the fiber layers are all relatively intact, with only a small area of damage. When the incident energy increase to 60 J, the interlayers between the fiber layers are also damaged to different degrees, which affects the load-bearing capacity of the laminate. When the incident energy reaches 80 J, the interlayers of both are penetrated. Although both show different degrees of damage at higher incident energies, the debonding area of FMLs-H is smaller compared to that of FMLs-C, which may be due to the interaction between the fibers mixed in, reducing the debonding area of the interlayers, thus enhancing the impact resistance of FMLs and providing a certain toughening effect. As the incident energy increases, the damage morphology of the FMLs, especially between the metal and fiber layers, also changes and eventually evolves into a circular shape.

A comparison of the conclusions obtain in this paper with those of Parnanen et al. [37] reveals that the experimental curves follow the same trend as their experimental results. The experimental results show that the hybrid fiber-reinforced magnesium alloy laminates have higher peak loads, but the pure carbon-fiber-reinforced magnesium alloy laminates have smaller displacements and better stiffness. However, only the mechanical response of FMLs under a low-velocity impact is investigated in this paper, and other mechanical properties such as tensile, compressive, flexural, and fatigue mechanical properties need to be investigated before a conclusion can be drawn on whether hybrid fiber-reinforced metal laminates are superior.

## 5. Conclusions

Compared with pure carbon-fiber-reinforced magnesium alloy laminates, carbon/glass hybrid fiber-reinforced magnesium alloy laminates have improved peak load and energy absorption by 5.1–8.5% and 3.7–5.7%, respectively, although the stiffness is slightly reduced. The use of hybrid fibers leads to better the impact resistance of FMLs.

The FEM results of FMLs were compared with the experimental results. It was found that the simulated values were slightly higher than the experimental values, and the differences between the peak load and energy absorption were 2.0–6.5% and 0.7–4.6%, respectively. The load–displacement curves and energy–time curves of the two are in good agreement, which indicates that the established finite element model can predict the experimental results better.

The damage morphology and damage modes of FMLs under low-velocity impact were observed and analyzed. When the incident energy is low, the damage mode of FMLs is petaling crack, and when the incident energy is increased, the damage mode of FMLs changes to circumferential crack.

At incident energies of 40 J, 60 J, and 80 J, the fiber damage area and debonding area of different fiber-reinforced magnesium alloy laminates become larger with the increase in incident energy. Compared with carbon-fiber-reinforced magnesium alloy laminates, hybrid fiber-reinforced magnesium alloy laminates have smaller damage area and better impact resistance and load-bearing capacity. An observation of the damage morphology of the fiber layer and the interlayer of FMLs reveals that the damage of the upper fiber layers is more serious compared to the lower fiber layers. Compared with the interlayers between the fiber layers, more serious debonding occurs between the fiber layers and the metal layers.

## Figures and Tables

**Table 1:** Information about FMLs.

Specimen Codes		Incident Energy	Ply Sequence
FMLs-C	FMLs-C1	40 J	Mg/C/C/C/Mg/C/C/C/Mg
	FMLs-C2	60 J	Mg/C/C/C/Mg/C/C/C/Mg
	FMLs-C3	80 J	Mg/C/C/C/Mg/C/C/C/Mg
FMLs-H	FMLs-H1	40 J	Mg/C/G/C/Mg/C/G/C/Mg
	FMLs-H2	60 J	Mg/C/G/C/Mg/C/G/C/Mg
	FMLs-H3	80 J	Mg/C/G/C/Mg/C/G/C/Mg

“Mg”, “C”, “H”, and “G” indicate magnesium alloy sheet, carbon fiber, hybrid fiber and glass fiber, respectively.

**Table 2:** The constitutive model parameters of AZ31B magnesium alloy.

A(MPa)	B(MPa)	n	*C*	m
172	360.73	0.45592	0.092	0.95

**Table 3:** The failure model parameters of AZ31B magnesium alloy.

$D_{1}$	$D_{2}$	$D_{3}$	$D_{4}$	$D_{5}$
−0.35	0.6025	−0.4537	0.206	7.2

**Table 4:** Mechanical properties of composite laminates.

Mechanical Property	Carbon Fiber	Glass Fiber
$X_{T}$ (MPa)	2093	567
$X_{C}$ (MPa)	870	241
$Y_{T},\;Z_{T}$ (MPa)	50	21
$Y_{C},\;Z_{C}$ (MPa)	198	83
$E_{11}$ (GPa)	128	45.6
$E_{22},\;E_{33}$ (GPa)	8.7	8.2
$G_{12},\;G_{13},\;G_{23}$ (GPa)	4.0	5.8
$S_{12},\;S_{13}$ (MPa)	104	65.2
$S_{23}$ (MPa)	86	42
$\nu_{12},\;\nu_{13},\;\nu_{23}$	0.3	0.3

**Table 5:** Gradual degradation model.

Failure Mode		Material Property Degradation Rules
Fiber	Tensile failure	$E_{11}=0.07E_{11}$
	Compressive failure	$E_{11}=0.14E_{11}$
Matrix	Tensile failure	$E_{22}=0.2E_{22},\;G_{12}=0.2G_{12},\;G_{23}=0.2G_{23}$
	Compressive failure	$E_{22}=0.4E_{22},\;G_{12}=0.4G_{12},\;G_{23}=0.4G_{23}$

**Table 6:** Material parameters of the adhesive layer elements.

E(MPa)			$\sigma^{m}(\mathrm{MPa})$			$G^{c}(N/\mathrm{mm})$			$\mathrm{Density}(\mathrm{kg}/m^{3})$
$E_{\mathrm{nn}}$	$E_{\mathrm{ss}}$	$E_{\mathrm{tt}}$	$\sigma_{n}^{m}$	$\sigma_{s}^{m}$	$\sigma_{t}^{m}$	$G_{n}^{c}$	$G_{s}^{c}$	$G_{t}^{c}$	ρ
2000	750	750	65	38	38	2	4	4	980

## Data Availability

Data are contained within the article.
